# Histologic evaluation of topical simvastatin effects on extraction sockets: A randomized controlled clinical trial

**DOI:** 10.34172/japid.025.3478

**Published:** 2025-06-11

**Authors:** Nasrin Faal Rastegar, Farzane Vaziri, Seyed Mostafa Mahmoudi

**Affiliations:** ^1^Department of Periodontics, Shahid Sadoughi University of Medical Sciences, Yazd, Iran; ^2^Department of Oral Maxillofacial Pathology, Shahid Sadoughi University of Medical Sciences, Yazd, Iran

**Keywords:** Dental socket preservation, Histology, Simvastatin

## Abstract

**Background.:**

The reduction of alveolar ridge volume after tooth extraction can be decreased through ridge preservation. According to previous studies, statin drugs induce osteogenesis. Therefore, this study aimed to evaluate the effect of simvastatin on the preservation and ossification of the alveolar ridge after tooth extraction.

**Methods.:**

In this single-center randomized clinical trial, 40 dental sockets in 40 patients were randomly divided into the treatment group (collagen with simvastatin) and the control group (collagen only). Histologic bone examination was performed under a light microscope two months after socket preservation at the time of dental implants. The predictable variable was using simvastatin in dental sockets. In the treatment group, collagen was used with simvastatin; in the control group, only collagen was used. The percentage of bone formation was the primary outcome, which was measured as the area of newly formed bone. In this study, inflammatory reaction, the amount of remaining bone substitute, and foreign body reaction were compared between the two groups. Covariates included age, sex, and tooth location. T-test was used for normally distributed data, while the Mann–Whitney test was used for non-normal data. *P*<0.05 was considered significant.

**Results.:**

The results showed that following eight weeks of simvastatin use in the treatment group, the percentage of new bone formation was significantly higher compared to the control group (treatment group vs. control group: 69.28±3.93 vs. 52.76±2.01; *P*=0.0001). No foreign body reaction and residual graft materials were observed in the treatment and control groups. Furthermore, the study showed an inflammatory reaction in only 23.5% of the samples in the control group (*P*=0.045).

**Conclusion.:**

Simvastatin significantly increased the formation of new bone in the dental socket in the treatment group.

## Introduction

 Tooth loss causes physiological and remodeling changes in the soft and hard tissues of the alveolar ridge, depending on multiple factors, including alveolar socket size, mucosal thickness, metabolic factors, and functional load.^1,2^ Bone resorption is inevitable, and implant placement is difficult unless steps are taken to preserve and regenerate it. Preservation of the alveolar ridge after surgery reduces residual ridge resorption and may improve implant placement from a functional and aesthetic viewpoint.^3-6^

 Autogenous bone is the most predictable material for augmentation processes.^7^ However, bone donor resources are limited, and autogenous graft harvesting is associated with complications such as bleeding, pain, and infection.^8^ Bone graft substitutes reduce the complications of the donor site and increase the implant’s success rate.^9,10^

 Statins, like simvastatin, are widely used drugs that reduce lipid levels. These drugs act through the mevalonate pathway. Several studies have shown that these drugs can regulate inflammatory responses through a mechanism independent of cholesterol reduction.^11^ Simvastatin is more desirable among statins since it can cross the cell membrane and has a shorter onset of action. It has the potential for osteoblast activation and osteoclast inhibition.They increase osteoblast differentiation by stimulating bone morphological proteins 2 (BMP-2).^12-15^ Administration of simvastatin is helpful in the healing of oral bone and cartilage.^16^

 Previous studies have reported the use of simvastatin in a variety of lesions, such as the subgingival area in periodontal lesions,^17^ class II furcation involvement,^18^ subgingival areas in smokers with periodontitis,^19^ gingival areas in patients with type II diabetes,^20^ and human maxillary sinus.^21^

 The potential of these drugs in soft tissue healing and TMJ arthritis has also been reported.^16^ Conflicting data exist on the use of statin in previous studies, and some factors like the method of administration and duration of exposure can influence the effect of simvastatin.

 This study evaluated and compared the percentage of bone formation in extraction sockets treated with simvastatin and collagen versus collagen alone.

## Methods

**Figure 1:**
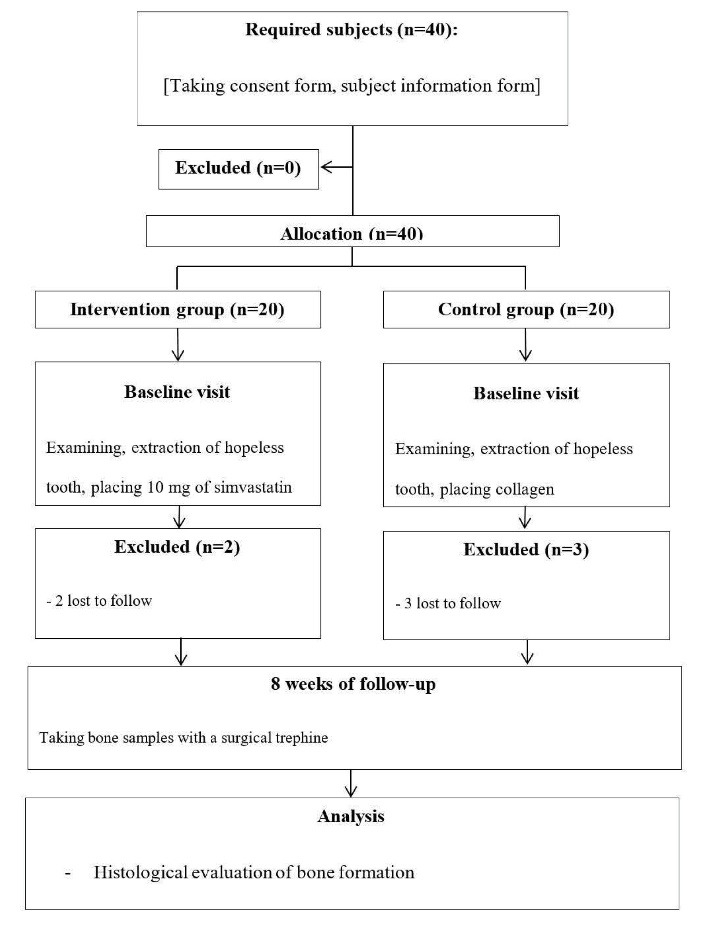
Flow diagram of patient selection

 In this single-masked sex and age-stratified, randomized clinical trial, 40 patients referring to the Periodontics Department of Dental School, Shahid Sadoughi University of Medical Sciences, Yazd, were selected (Figure 1). All the patients enrolled in this study underwent the extraction of hopeless teeth and were divided into treatment and control groups. The present study was conducted using the principles of the Helsinki Declaration and Consort Guideline 2010 (Supplementary file 1). The study was approved by the Ethics Committee of Shahid Sadoughi University of Medical Sciences (IR.SSU.REC.1397.120) and was registered in the IRCT registry with the identification code IRCT20171015036782N6.

###  Inclusion criteria

 Patients with hopeless premolar and molar teeth and candidates for implant placement were included.

###  Exclusion criteria 

 Patients with periodontitis, systemic diseases like diabetes, pregnancy, a history of radiotherapy and steroid drugs, smoking, and a history of the systemic use of statins were excluded.

###  Intervention

 Patients were divided into treatment and control groups according to the randomized number table by an assistant blinded to the details. The surgeon was aware of group allocation, but patients and the pathologist were blinded to group assignment. Before surgery, mouth rinsing was performed with 0.2% chlorhexidine gluconate mouthwash for 1 minute. After local infiltration anesthesia with 2% lidocaine and 1:100,000 epinephrine, a full-thickness mucoperiosteal flap was elevated, and a hopeless tooth was extracted. The intact dental socket wall was curetted and rinsed with a normal saline solution. In the treatment group, 10 mg of simvastatin (one 10-mg tablet in powdered form) in combination with collagen was placed in the extraction socket. In contrast, only collagen was placed in the control group. The socket was covered with 10*10-mm acellular dermal allograft (Cenomembrene, Hamanand Saz Baft Tissue Regeneration Corporation, KFZ, Iran), and the flap was closed with 3-0 vicryl suture to achieve primary closure. The next session was scheduled two months after extraction, in which bone samples were taken using a 3.5-mm surgical trephine from the middle part of the socket for the histologic examination. The bone samples were fixed in 10% formalin solution for 48 hours and decalcified in formic acid for one week. Histologic longitudinal sections measuring 5 µm were stained with hematoxylin and eosin (H&E). In each sample, 5 fields with the highest bone density were selected under × 400 magnification, and the image was taken with a camera attached to a microscope. ImageJ software was used to examine the images.

**Figure 2:**
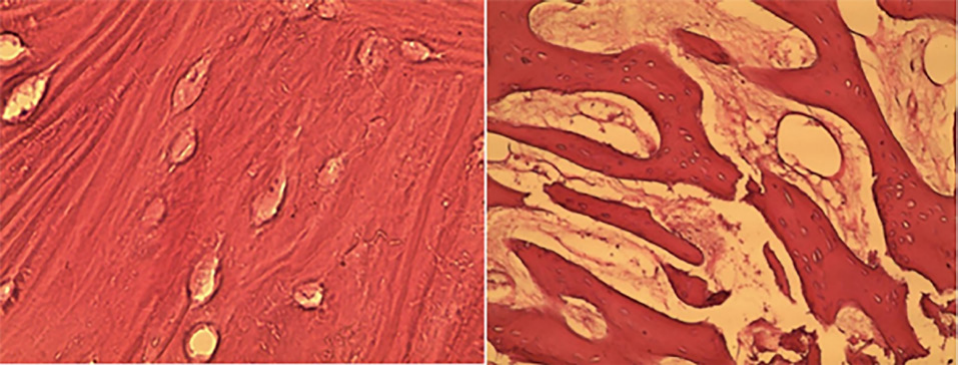
Osteogenesis in the treatment and control groups at 8 weeks; left side: treatment group, right side: control group (HE × 400). Arrowheads: bone trabecula

 Additionally, foreign body reaction, inflammatory reaction, and histological features of the bone substitute material were evaluated. Figure 2 shows histological sections of osteogenesis.

**Table 1:** Patient’s demographic characteristics in the treatment and control groups

**Demographic characteristics**	**Treatment group**	**Control group**	***P*** ** value**
Number, n%	18	17	
Male, n%	13 (72.2%)	7 (41.2%)	0.64
Female, n%	5 (27.8%)	10 (58.8%)	0.64
Age/year	42.50 ± 14.52	34.47 ± 15.02	0.169

Pearson’s chi-squared test

**Table 2:** The mean percentages of bone formation

**Group**	**Number**	**Mean percentage of bone formation**	**SD**	***P*** ** value**
Treatment	18	69.28	3.93	0.0001
Control	17	52.76	2.01	

T-test.

###  Primary and secondary outcomes

 The primary outcome of this study was the amount of bone formation, which was measured as the percentage of the bony tissue area in the total tissue area. The secondary outcomes were the percentage of inflammatory reactions, remaining bone substitutes, and foreign body reactions in the total tissue area.

###  Data collection method

 The collected data included bone formation, foreign body reaction, a remnant of a bone substitute, and inflammatory reaction. The formation of new immature bone was calculated as a percentage of surface area in the histologic section. The foreign body reaction, defined as granulomatous inflammation and the formation of foreign body granuloma, epithelioid macrophages, and multinucleated giant cells, can be seen in histopathological examination with H&E staining. Residual graft materials were seen as amorphous material in the histologic section. The inflammatory reaction was evaluated as lymphocyte infiltration in each section.

###  Sample size calculation 

 Considering a significance level of 5%, a power of 80%, and according to the results of a previous study,^22^ to achieve a significant difference of at least one unit in the mean amorphous bone while anticipating a standard deviation of S = 0.6, 20 subjects were included in each group.

 SPSS 23 was used for statistical analysis.

###  Statistical analysis

 Data were measured as mean ± standard deviation and evaluated via the Kolmogorov-Smirnov test to assess normal distribution. Normally distributed data were compared via t-test, while non-normally distributed data were compared via Mann–Whitney and Fisher’s exact tests. The statistical significance level was considered at *P* < 0.05.

## Results

###  Baseline characteristics

 Of the 40 patients enrolled in the study, 35 completed this research. The mean age was 42.50 ± 14.52 years in the treatment group and 34.47 ± 15.02 years in the control group. The treatment group consisted of 13 males and 5 females, with 7 males and 10 females in the control group. Eighteen dental sockets were treated with simvastatin and 17 without it. There was no significant difference in age and sex between the two groups (Table 1).

###  Primary outcome

 The mean amounts of bone formation in the treatment and control groups are presented in Table 2. According to Figure 2, bone formation in the treatment group was significantly higher than in the control group. Multiple linear regression was used to eliminate the confounding factors of age and sex. These analyses showed that after age and sex matching, there was a statistically significant difference between the treatment and control groups.

###  Secondary outcome

 Neither the treatment nor the control group exhibited a foreign body reaction and bone substitute remnant. Therefore, the two groups had no significant difference in histological characteristics of foreign body reaction and bone substitute remnants.

 No inflammatory cell infiltration was observed in both groups, except that 23.5% of the control group showed mild chronic inflammatory reactions.

## Discussion

 Alveolar ridge preservation, synonymous with socket preservation, was first described as bone maintenance in 1982.^23^ The shape and volume of the alveolar process are determined by the presence or absence of teeth and their inclination in the bone.^1,24^ According to controversy regarding material choice in socket augmentation, decision-making on selecting materials in socket grafting is important. As we have limited donor sites for autogenous bone harvesting and its associated morbidity, several studies recommended using an alternative material as a substitute for autogenous bone.^23^ Given statins’ antibacterial, anti-inflammatory, and osteopromoting properties, their topical use is recommended as adjunctive therapy to surgical and nonsurgical periodontal treatments.^25,26^ Given the importance of bone preservation during tooth extraction and the reduction in bone resorption after tooth extraction, the specific aim of this study was to evaluate the effect of simvastatin on bone regeneration in human dental sockets after tooth extraction, which was defined as the proportion of newly formed bone.

 In the present study, a comparison between the two groups showed that the rate of bone formation was higher in the collagen and simvastatin group compared to the other group, consistent with previous studies.^19,22,27^ Wu et al’s^27^ study indicated the effectiveness of preserving the alveolar bone of the dental socket after the topical use of simvastatin. Unlike the present study, the study above was an animal study and cannot be reliably generalized to humans. Additionally, in the study above, polylactide-co-glycolide acted as the carrier for simvastatin, unlike the collagen in our study. The follow-up duration was two months, and the treatment and control groups differed in both studies.

 Rao et al^19^ performed a radiographic evaluation and determined clinical parameters after local delivery of simvastatin in smokers with chronic periodontitis. In this study, clinical parameters such as probing depth and clinical attachment loss were evaluated in 6- and 9-month follow-ups. They injected simvastatin gel into the periodontal pockets that had vertical bone defects. The simvastatin group sites achieved significantly greater vertical defect fill compared to the placebo group. Their methods were completely different from the present study in that the simvastatin application was different from the present study. After two months, the bone quality and quantity of the dental socket were assessed histologically, and smokers were excluded. However, both studies are valuable as they were performed on human subjects. Tanabe et al.^28^ showed fluvastatin’s potential for bone regeneration in an animal study. Unlike the present study, the above study was conducted outside the oral environment. Yaghobee et al^21^ evaluated the efficacy of simvastatin administration with bovine bone material to augment the human maxillary sinus in a split-mouth design. This study showed that the amount of newly formed bone and residual particles did not differ significantly between the two groups, even though the surgical site was the maxillary sinus and the follow-up period was 9 months. Diniz et al^29^ studied the effect of the local application of simvastatin (10 mg) on bone regeneration after surgical removal of bilaterally impacted mandibular third molars. The radiographic results favored simvastatin, indicating that local application of simvastatin could be a cost-effective and simple way to accelerate osseous regeneration. Koç et al^30^ evaluated the combination of melatonin and simvastatin on bone regeneration in rats. They demonstrated that a combination of melatonin and simvastatin had a synergistic effect on bone regeneration. The methods used in the present study were similar to those of Sezavar et al^22^ However, the differences are that our study’s design was not split-mouth, and the treatment and control groups had different and separate models, which are the limitations of our study. The different dosages of simvastatin in both studies are noticeable. Also, our study evaluated the presence or absence of foreign body reactions and the amount of residual graft materials. Only 23.5% of the control group subjects in our study showed an inflammatory reaction.

 Contrary to our study, the samples of some studies were animal models.^27,28,31^ Histological evaluations were carried out in the present study in contrast to radiographic and clinical evaluations in other studies.^20,31-33^ Histological evaluations of the present study could assess bone quality and quantity more accurately. Another limitation of our study was the flap reflection, which can influence bone resorption. It should be noted that this procedure was done in both the treatment and control groups, and both groups were influenced by it.

## Conclusion

 In conclusion, the present study’s findings showed that simvastatin use in tooth sockets resulted in higher bone formation compared to the healing of the tooth socket with collagen alone. Therefore, it can be an effective substance during the healing period in tooth sockets after extraction to gain more mineralized bone.

## Competing Interests

 The authors declare that they have no competing interests.

## Data Availability Statement

 The data from the reported study are available upon request from the corresponding author.

## Ethical Approval

 The present study has the code of ethics IR.SSU.REC.1397.120 of Shahid Sadoughi University of Medical Science, Yazd, Iran.

## Supplementary File

 CONSORT 2010 checklist of information was used to include when reporting a randomized trial.
